# Advances in Micro-Bioreactor Design for Organ Cell Studies

**DOI:** 10.3390/bioengineering5030064

**Published:** 2018-08-10

**Authors:** Carl-Fredrik Mandenius

**Affiliations:** Division of Biotechnology, IFM, Linköping University, 581 83 Linköping, Sweden; carl-fredrik.mandenius@liu.se; Tel.: +46-13-28-1000

The engineering design of microbioreactors (MBRs) and Organ-on-Chips (OoCs) has advanced considerably in recent years [1,2]. The term MBR originally referred to the bioengineering methodology of performing biological reactions in micro-scale reactor devices; the term OoC refers to the recreation of organs and tissues from the human body on or in a miniaturized device with a smaller volume than the original organ and with body-like fluids streaming around the cells in an in-vivo-like fashion. Despite this difference, many of the basic engineering principles coincide in the design of MBRs and OoCs. However, there are also striking differences in the design requirements due to the purposes of use.

In bioprocess development, the main aim of the use of MBRs is to accelerate the development work of new bioprocesses with microorganisms or mammalian cells as production organisms [3]. The culture of the manufacturing process is typically scaled down to 1–10 mL volume in the MBR, and critical process parameters and media composition are systematically optimized. The increased yield and productivity of the large-scale process can be reached at a much earlier stage in the development process with this approach. Commercial MBRs with >100 parallel MBR units are now on the market.

The aim of OoC devices is to facilitate the study of organ cell assemblies in vitro under conditions that recreate in vivo conditions of the organ in the body for recapitulating time-related cellular behavior [4,5]. An OoC device allows for the observation of cellular effects when exposed to drugs or other chemicals. This allows for the assessment of compounds’ effects at subcellular and multicellular levels. Successfully applied, this supports the investigation of safety pharmacology.

This special issue addresses these diverse aspects of MBR design in nine expert contributions where a variety of cells and tissues are used with various aims and ambitions.

The fundamental design challenges in MBRs are highlighted in two review contributions [6,7]. The similarities of the design of different MBRs, despite purposes, lead to a general design methodology for MBRs where functionality drives the design [6]. The engineering-based design of MBRs and OoC devices can take advantage of established design science theory, in which a systematic evaluation of functional concepts and user requirements takes place. The review compares how such common conceptual design principles are applicable to MBR and OoC devices. The complexity of MBR design, which is exemplified for scaled-down cell cultures in bioprocess development and drug testing in OoCs for the heart and the eye, is discussed and compared with previous design solutions of MBRs and OoCs from the perspective of how similarities in understanding design from functionality and user purpose perspectives can more efficiently be exploited. The review can serve as a guideline and help the future design of MBR and OoC devices for cell culture studies.

Seldon and Fuller [7] further address the challenges of introducing organ and tissue cells in MBRs for understanding normal and pathological physiology. The differences and the constraints of the physiological environment that influence the design are highlighted. This review considers the key elements necessary to enable bioreactors to address the critical areas associated with biological systems.

The use of MBRs as tools for investigating tumor models is highlighted in two research studies [8,9]. Kuhlbach and colleagues [8] have studied tumor extravasation on a chip. Their device consists of three different parts, containing two microfluidic channels and a porous membrane sandwiched in between. In contrast to many other systems, this device does not need an additional coating to allow endothelial cell (EC) growth, as the primary ECs used produce their own basement membrane. The ECs in their device showed in vivo-like behavior under flow conditions. These results suggest that the new device can be used for research on molecular requirements and conditions and the mechanism of extravasation and its inhibition.

Toh and colleagues [9] have developed a microfluidic-based culture chip to simulate cancer cell migration and invasion across the basement membrane. In this microfluidic chip, a three-dimensional (3D) microenvironment is engineered to culture metastatic breast cancer cells in a three-tumor model. The chip is useful for drug screening due to its potential to monitor the behavior of cancer cell motility, and, therefore, metastasis, in the presence of anti-cancer drugs.

Investigating effects of drug compounds on organ cells in in vitro microfluidic models has been mentioned recurrently to fill the need in the pharma industry for more efficient drug testing. In a study by Christoffersson and colleagues [10], a 3D model with cells arranged in spheroids is shown to be a valuable tool to improve physiologically relevant drug screening. In this article, it is shown how the number of cells growing out from human-induced pluripotent stem cell (hiPSC)-derived cardiac spheroids can be quantified to serve as an indicator of a drug’s effect on spheroids captured in a microfluidic device.

Freyer and colleagues [11] demonstrate another approach with the same purpose with liver cells. They investigated the response of primary human liver cells to toxic drug exposure in a miniaturized hollow-fiber-based bioreactor. The results validate the suitability of the microscale 3D liver construct to detect hepatotoxic effects of drugs in a perfused human in vitro culture platform.

In another MBR setup, Wrzesinski and Fey [12] carry out an in-depth study of hepatocytes metabolism. They describe basic principles and how they are regulated so that they can be taken into consideration when microbioreactors are designed. They provide evidence that one of these basic principles is hypoxia, a natural consequence of multicellular structures grown in microgravity cultures.

Aspects of fluid dynamics in MBRs are addressed by Tajsoleiman et al. [13]. Due to the sensitivity of mammalian cell cultures, understanding the influence of operating conditions during a tissue generation procedure is crucial. In this regard, a detailed study of scaffold-based cell culture under a perfusion flow is presented with the aid of mathematical modelling and computational fluid dynamics (CFD). The simulation setup provides the possibility of predicting cell culture behavior under various operating conditions and scaffold designs.

Another important aspect of MBR design is oxygen distribution. Fernandez et al. [14] demonstrate the use of oxygen sensors to measure the oxygen consumption rate of several variants during the conversion of styrene (substrate) to 1-phenylethanediol (product). The oxygen consumption rate allowed for distinguishing the endogenous respiration of the cell host from the oxygen consumed in the reaction. Furthermore, it was possible to identify the higher activity and different reaction rate of two variants relative to the wild-type NDO.

All together, these nine contributions reflect state-of-the-art aspects of MBR design and highlight the inherent potential and strengths of the concept of MBRs for organ cell studies.
